# Treatment of Loose Teeth Due to Inflammatory Degeneration of the Gums and Alveolar Process*Read in the Section of Stomatology of the American Medical Association, at the Sixty-Fourth Annual Session, held at Minneapolis, June, 1913.

**Published:** 1914-02-15

**Authors:** Joseph Head

**Affiliations:** Philadelphia, Pa.


### TREATMENT OF LOOSE TEETH DUE TO INFLAMMATORY DEGENERATION OF THE GUMS AND ALVEOLAR PROCESS.*

JOSEPH HEAD, M. D., D. D. S., PHILADELPHIA, PA.

The disease of pyorrhea alveolaris, in spite of its name, is not always associated with a flow of pus from the infected tooth-sockets. As a matter of fact, pus does not appear in 25 per cent. of the cases. The main symptoms are inflammation of the peridental membrane, bleeding of the gums at the slightest touch and a tendency for the teeth to elongate and be sensitive to mastication. Finally, the gums separate from the roots of the teeth, forming pockets in which salivary calculus and infection cause such centers of irritation and disintegration that the destruction of the teeth involved is only a question of time unless the tartar is removed from the roots, the mass of infection destroyed and

*Read in the Section of Stomatology of the American Medical Association, at the Sixty-Fourth Annual Session, held at Minneapolis, June, 1913.

the gums stimulated to reattach themselves to the roots of the teeth.

A single blind fistula from an infected root may cause the most profound nervous depression, which, being chronic, will be recognized only by its disappearance with the cure of fistula.

Osler, Goadby, Daland, Hunter and Billings, I believe, have not overstated the bodily ailments that time will show to be directly traced to mouth infections. That mouth infection is, in a great majority of cases, associated with Bright's disease, diabetes, cardiac, hepatic, renal and stomach disorders has been noted for a long time, but within recent years it has been shown that these disorders are, in many instances, ameliorated and, at times, entirely eradicated by the removal of the infecting foci from the mouth. My experience in the treatment of pyorrhea alveolaris with its associated tooth infections accords with these findings.

The development of medical science has now placed on the dentist not only primarily the repair and restoration of the teeth that they may look well and give efficient power of mastication, but the responsibility of deciding whether or not patients shall be inflicted with a long list of diseases that owe their origin and existence to infections within the teeth and the adjacent tissues. This question of autotoxemia has been a great shock to the prejudice of the medical world at large, but to the dental world, trained more along purely mechanical lines, with little or no bacterial knowledge, the question of autotoxemia is no less than revolutionary. It is as though cabin-boys had suddenly awakened to find that they had been steering the ships of the human fleet, without charts or knowledge of navigation, over rocks, whirlpools and mines, all the time blaming unavoidable fate for the death and disaster, when the wrecks, deaths and disasters were directly caused by their own well-meaning, blundering hands. The medical world at large cannot escape this indictment, but Dentistry, inasmuch as she recommends appliances and fixtures that cannot be cleansed, that obviously may cause and perpetuate toxemia, is overwhelmed by the contemplation of the long list of preventable diseases and the gruesome toll of deaths that her well-meant efforts may have unwittingly occasioned. The manifestly undisputed fact that the removal of non-cleansable crowns and bridges have caused the rapid disappearance of heart murmurs, violent nervous derangements, duodenal ulcer, anemia, etc., is a convincing proof of what wholesale disease had been caused by the mechanical appliances now generally recommended.

When we come to pyorrhea and pyorrhea pockets, which the great majority of dentists class as incurable, we have come to the great divide, for those dentists who shall control and cure this great scourge shall take their places in the dentistry of the future, and those who neglect this important source of human disease shall be relegated to the spheres of those who unwisely attempted that which they were unfitted to accomplish; for the cure of pyorrhea alveolaris has been more or less successfully accomplished for the last fifteen or twenty years.

While, as previously stated, pyorrhea is associated with various disturbances of which it may be the primal and continuing cause, such as gout, Bright's disease, diabetes and cardiac, hepatic and renal disorders, such specific disease as syphilis, gonorrhea and tuberculosis, by their debilitating effect on the tissues of the mouth may so lower the vitality of the gums that infections that otherwise would be repudiated, penetrates the peridental membrane and by a subtle toxemia still further depress the general vital forces. This infection having penetrated the gums, forms a small abscess that partly strips away the peridental membrane from the tooth-neck, forming a pocket, which once formed continues the disease even after the general systemic disorder may have been conquered. It is probable that some one of the numerous passing diseases, such as typhoid, meales, grip, or even a bad cold, has temporarily lowered the physical resistance of the gum tissue to the point of permitting the ever-present infection to penetrate and form the self-perpetuating pockets, thus leaving the pyorrhea to continue without apparent systemic cause.

If the systemic cause has passed and the pyorrhea has not created a new one, the disease may perhaps be successfully treated by local means and a permanent cure hoped for; but if the pyorrhea is associated with serious general disorder, local treatment, important as it is, can be held only as a palliative and must be supplemented by judicious systemic treatment. The most important systemic treatment in the cure of pyorrhea consists in the discriminating use of vaccines that will produce antibodies in the blood as supplementary adjuncts to the proper local treatment; and yet, important and valuable as the vaccine treatment unquestionably is, without the proper local treatment that removes infecting foci and stimulates the gums to heal and re-attach themselves to the roots, vaccine treatment can only reduce the severity of the symptoms; it cannot affect a cure.

About seven years ago it was my good fortune to discover that commercial hydrofluoric acid would disintegrate tartar with no microscopic action on either cementum or enamel of an extracted tooth. The poisonous action of this acid precluded its use as a therapeutic agent to be applied to the gums. Through an extensive series of experimentation it was proved that a 23 per cent. solution of ammonium bifluorid (and acid salt of hydrofluoric acid) will disintegrate the tartar on a tooth as readily as hydrofluoric acid itself and also leave the tooth apparently unsoftened. The following is a safe, practical method for its manufacture: Forty c. c. of commercial hydrofluoric acid should be placed in a wax bottle and 27 gm. of chemically pure ammonium carbonate slowly added, a small lump at a time, the bottle being closed after each addition to prevent undue escape of fumes. When this mixture is allowed to rest for twenty-four hours it is ready for use. The temperature of the mixture during manufacture should not exceed 60 degrees Fahrenheit. The solution of ammonium bifluorid thus obtained gives excellent therapeutic results. Any variation in the strength of the acid made by this method seems to be of no importance chemically.

The solution of ammonium bifluorid may be injected once or twice a week into pockets around loose teeth, but care must be taken not to inject it into fresh cuts, as such procedure will cause great pain. The patient should spit without rinsing the mouth with water, the saliva acting as a protection against irritation. After one or two injections the soreness and inflammation will largely disappear, and even the general symptoms of toxemia will sometimes be found to have abated. The tartar scale that could not be easily and painlessly removed at the first two treatments will now tend to be so loosened that its thorough removal by scalers will be easy for both patient and dentist. After four or five applications, one week apart, black scales that have escaped the instruments will sometimes be found floating loose in the pockets, so that they can readily be picked out; finally, the root will become as smooth as velvet to the touch of an instrument. When this stage has been reached it will be found that the scalers cannot be so deeply carried into the pyorrhea pockets as was easily possible in the beginning. To do so causes pain and a free flow of blood. This indicates that new granulations are forming, which should not be ruthlessly broken up either by instrumentation or by injection of the bifluorid into them. Once each week the solution should be allowed to flow gently into the pockets, a procedure which results in continuous healing and is not productive of pain. Careful exploration for tartar on the root within the pocket is always advisable. Re-attachment of the gum to the root, however, can be accepted as assurance that that portion of the root has been thoroughly cleansed. The scaling in that place should then be discontinued, while the weekly applications of the bifluorid alone should be used. If, however, at the end of two or three months any of the pockets have not entirely healed they should be re-explored with scalers and the treatment repeated as for a new case, although by this time most of the original pockets will have disappeared, and where they existed the gums, with a slight contraction at the neck, will be found firmly adherent to the tooth. Teeth that have lost more than half of their gum attachment have become, under this treatment, firm and comfortable to the action of normal mastication.

Let us now consider some of the other means of local treatment in the cure of pyorrhea alveolaris. Loose teeth may be splinted together so as to give immobility, but this should always be done in such a manner as not to prevent thorough cleansing of the gum around their necks. Any malocclusion should be remedied so that loose teeth may be relieved of the excessive pressure of mastication due to thickening of the inflamed peridental membrane. When a pocket has approached an apical foramen near enough to infect the pulp, the pulp should be destroyed and the root canals thoroughly sterilized and filled. This should be especially looked to in the case of molars in which the pocket extends through the bifurcation and down to the tip of one of the roots. It is sometimes excellent practice to cut the crown midway through the grinding surface through the juncture of the roots, finally extracting the diseased root with its side to the crown. This exposes the other root or roots so that they can be filled and cleansed as easily as a bicuspid. With upper molars, if the palatal root is necrosed, this operation is most satisfactory as it leaves the mouth to all appearances unchanged, and works for greater stability and more rapid recovery by not forcing the remaining root or roots to stand more than their fair share of mastication.

An excellent mouth-wash for use at home consists of a saturated aqueous solution of sodium silicofluorid which amounts to a 0.61 per cent. solution. This, when held for a minute in the mouth, morning and evening, and freely swashed for at least a minute through the teeth is a great assistance in relieving inflammation of the gums. Like the bifluorid it seems to restrain the deposition of tartar on the teeth, except in about 1 per cent. of cases, in which it seems to increase it slightly. But even in these cases the gums heal promptly and the superficial deposit is easily removed from the teeth with brush and pumice. The disagreeable flavor of the solution can be disguised by adding an equal amount of sodium chlorid or a judicious quantity of aromatics.

An efficient tooth-powder that will liberate enough free oxygen to make from 40 to 50 drops of a 3 per cent. peroxid solution for every 10 grains used on the brush in the mouth is as follows:

| | |
|---|---|
| Magnesium peroxid (200 mesh sieve) | 60 parts |
| Sodium perborate | 30 parts |
| Castile soap and flavoring | 10 parts |

This can be used morning and evening for brushing the teeth and should be swashed around the interstices for a full minutes before being ejected from the mouth. Without thorough cleansing and brushing of teeth and gums morning and evening antiseptic washes will, however, be of no avail. Since the tooth-brush cannot cleanse between the teeth, these surfaces should be swept free from bacterial deposits with floss-silk morning and evening, and the teeth and gums thoroughly brushed with strokes not less than an inch and a half long, and rotary wherever possible. The brush should be small, not over an inch and a half long, the bristles not over a quarter of an inch, and narrow, so that when the mouth is partly opened the brush can be placed between the ramus of the jaw and the third molars. Most brushing does not extend beyond the spring of the bristles, which instead of giving bristle friction merely pivots the bristles without cleansing. The upper and lower third molars more frequently decay and are subject to pyorrhea alveolaris simply because they are not cleansed. Structurally, they are not weaker than any other teeth. At each visit of a patient it is most essential that the necks of all the teeth be examined for bacterial plaques that may have been accumulating undisturbed since the last visit. The patient should be shown what movements of the brush are necessary for their removal, for a final test of a method of brushing the teeth and gums is, does it clean away the bacterial plaques? Ninety-nine out of a hundred cleanly people never brush the thick bacterial plaques from their third molars, or, as a matter of fact, from half the other teeth, simply because they have never been taught how. Gums as well as teeth should be thoroughly brushed twice a day of all bacterial plaques. Observation on my patients proves that healthy gums are no more injured by vigorous brushing than is the skin adjacent to the finger-nails. Inflamed soft gums will unquestionably be made sore for the first week or two, but persistence on the part of the patient and assistance on the part of the dentist in touching up the sore spots with silver nitrate will soon strengthen the gums to almost any friction the tooth-brush can give them. The question of vigorous cleansing and massaging the gums with the brush is not only a question of removing external films of infection, but is also for the purpose of producing an auto-inoculation that will create antibodies in the blood for the purpose of combating the disease, a vigorous message of the parts causing a local hyperemia, which enables the antibodies in the blood to come into more intimate contact with the infecting bacteria. This is a most important phase in the cure of pyorrhea and one that has not been sufficiently emphasized. The formation of antibodies for the cure of pyorrhea is a means of eradicating pyorrhea from the system. The auto-inoculation caused by vigorous gum brushing combined with the judicious use of carefully prepared vaccines have given results of a systemic improvement that are little less than marvelous.

Medical literature is so full of the reports on successful vaccine treatment for mouth infection that they cannot but convince the most skeptical that there must be value in the treatment. Goadby's work in this field is deserving of great credit.* Cummins has published a recent article on this subject wherein Goadby's methods are followed. The work of Leary and his associates is more in accordance with modern

*Cummins, R. C.: Jour. Vaccine Therap., March, 1913, p. 59.

bacteriologic methods, however, and their reports are more in accord with the bacterial findings of this paper.

About two years ago I began the vaccine treatment as an adjunct to my local pyorrhea treatment. I was decidedly skeptical because pyorrhea does not seem to be caused by a specific bacterium, but may be caused by any one of several species, or various combinations of these. Nevertheless, from the very start such increased improvement locally and systemically was obtained that the great value of the treatment could not be doubted. The results in almost every case showed a consistent healing of the pockets and a disappearance of infection from the gums. In about 50 per cent. of the cases there was a distinct improvement in the general physical condition, which, as before stated, being chronic was sometimes made more apparent by its absence than its presence. In the forty cases that are used as the basis of this report, the streptococcus, staphylococcus, *Bacillus influenzae*, pneumococcus, *Micrococcus catarrhalis*, diphtheroids, Friedlander bacillus and an occasional unidentified bacterium were used in the vaccines. The *Streptococcus viridans* appeared in about 25 per cent. of the cases. That the vaccines for pyorrhea alveolaris for forty cases contained such a consistent combination of similar germs for so many cases with so comparatively few unidentified strains may be partly due to the method used in collecting the specimens so as to exclude the incidental or extraneous flora of the mouth. My method will now be described.

When the infected area appeared at the tip of a root in which the pulp had died and the root canal had been filled, the culture was always taken through the root canal, which was drilled out with a fine sterilized piano-wire drill until the end was nearly reached. The canal was then sterilized with phenol (carbolic acid), wiped dry with cotton and then blown out with hot air. When this had been accomplished and the tooth had been carefully guarded with a napkin to prevent infection from the mouth, another sterilized drill was passed down to the end and then plunged through the tip into the infected area. This was then streaked over the blood-agar in the ordinary manner. At times, however, the infected area appeared near the tip of a root or roots in which the pulps were alive. In this case the root-canal method of obtaining the specimen was not feasible, so the following method was substituted: The mucous membrance over the indurated infected spot was cocainized and a thin cautery plunged down to the bone. A sterilized bone-drill was then passed through the outer plate of the alveolar process and the patient dismissed for two days. On his return the opening in the gum, on being protected with a napkin, was cauterized with pure phenol and then wiped dry. Then the sterilized point of a small sterilized platinum-pointed glass syringe or platinum spear was inserted into the bony opening made previously by the drill, and a small drop of bloody fluid extracted, which with due care was transferred to the blood-agar medium. This material was supposed to contain the bacteria that had gathered for the purpose of preventing reorganization of the tissues.

To take a specimen from a pyorrhea pocket the following method was used: The neck of the tooth was first carefully washed with 95 per cent. alcohol so as to remove all outside bacteria as much as possible. The tooth was protected from mouth infection by a napkin, and then a small cup-shaped spear of thin platinum was heated to a cherry red and plunged to the bottom of the pocket. Such a procedure it was thought would get not only the pus but also a slight amount of blood from the walls of the cavity. It was thought that the hot platinum would kill any extraneous flora, while the cooled metal would carry to the blood-agar only the germs directly responsible for the disease in question. It must not be forgotten that pus is sometimes sterile, while the true cause of infection may lurk within the walls of the abscess from which the material is being obtained.

The vaccines were then made up so as to contain all the germs found, except the spore formers and the anaerobes that were not grown. While the bacteria of these classes may sometimes play a role in the infection the results of vaccine treatment indicate that in these cases their role is a minor one. The staphylococci were made to give 300 million to the cubic centimeter. Streptococci, diphtheroids, pneumococci, *Micrococcus catarrhalis, Bacillus influenzae* and unclassified bacteria were put in so that each would show 50 million to the cubic centimeter. In ordinary cases of chronic pyorrhea the initial dose is 75 million staphylococci and 12 million of each of the other bacteria, and the dose is steadily raised according to the reaction at the site of inoculation and the general systemic response. If the patient showed exceptional frailness or the inflammation was exceptionally acute, the initial dose was reduced to 37 million staphylococci and 6 million of each of the other bacteria. These doses were generally given a week apart in the arm, or, in thin patients, in the back.

I have not used the opsonic index in the treatment of pyorrhea alveolaris. I feel that clinical symptoms have been a sufficient guide. An interesting observation which I have not seen mentioned elsewhere is the occasional persistence of the induration which forms at the point of injection. It has appeared to me that this can be due only to a lack of digestion and absorption of the bacterial bodies. So long as the induration markedly persists we have an indication that the potentialities of the vaccine injection have not been exhausted. Therefore, further injections may be temporarily withheld or smaller doses given while absorption in the seats of induration is stimulated by massage. This method gives excellent results, and tends to minimize the danger pointed out by Allen,[3] when excessive reaction is caused by doses which may have been perfectly well borne on several previous occasions. As Allen says, however, it should always be borne in mind that, if the systemic reaction lasts over twenty-four hours, it is wise to increase the interval between

3 Allen: Vaccine Therapy and Opsonic Index, Blakiston, Philadelphia, 1913. p. 112.

the doses, and a larger dose should not be given especially if the patient makes good progress on the smaller dose.

If there is a tendency to the rapid formation of creamy tartar deposists on the teeth prior to vaccine treatment, it will be noted that, as the antibodies are formed and the gums show signs of healing, the tartar will be deposited much less rapidly and that the tartar deposited is of a more solid, removable nature and does not tend to burrow under the gum margins. This change in the deposition of tartar I have finally come to regard as a distinct symptom of the successful progress of the vaccine inoculations.

When a pyorrhea pocket shows sudden signs of inflammation during treatment it is always wise to open it surgically with a drill along the root to be sure that there is no back-pressure of pus, and that the antibodies have full opportunity to enter the seat of infection for the purpose of aiding in the cure; for above all things it should be remembered that the vaccine treatment can be successful only when accompanied by judicious local treatment of a surgical and therapeutic character. This applies to all foci of infection whether in the gums or in the impaction of a bowel or anywhere else in the system. It is imperative, therefore, that there should be sympathetic cooperation with the family physician, whose intimate knowledge of the patient and whose careful diagnosis of foci of infection other than those found in the mouth will greatly increase the percentage of successes and add to the permanence of the cures.

Allen[3] speaks of the value of citric acid in 30-grain doses three times a day for the purpose of softening the lymph-wall around the foci of infection by reducing the agglutinative power of the blood. I have found this treatment of service, but give it in the form of an ounce of lemon juice three times a day, which is the equivalent of about 34 grains of citric acid.

I shall now report a few detailed cases.

*Cases 1 and 2.*—A young man with pronounced pyorrhea

---

3. Allen: Vaccine Therapy and Opsonic Index, Blakiston, Philadelphia, 1913, p. 112.

and several fistulas in the gums came to me so despondent that he thought he was going to die. Local treatment caused improvement, but two of the fistulas would not heal. An autogenous vaccine was made in March, 1912, from material obtained from the worst fistula. It contained only a single strain of streptococcus. After four injections, a week apart, the patient gained in weight and then started slowly to lose weight, but as he felt better and the gums improved, the treatment was continued until June, when there was a great improvement in the gums and both fistulas had healed. In the fall the patient returned and, as he and his family physician said that the vaccine had done him so much good, I made another vaccine from a small pyorrhea pocket and obtained four strains of streptococci and a *Staphylococcus aureus*. This treatment caused a complete healing of the mouth so that it was apparently absolutely healthy; the patient's skin cleared, he gained weight and felt, as he said well. The mixed vaccine seemed most effective.

He begged me to give the vaccine treatment to his wife who also was suffering from pyorrhea. She had almost the same experience, except that she did not gain weight (her weight was normal), but her mouth showed great improvement and she slept better and felt a great lessening of nerve tension.

*Case 3.*—A young woman was vaccinated with a Gram-negative bacillus, 50 million to the cubic centimeter, with some improvement, but not marked. Later a second vaccine was made of *Staphylococcus aureus* and *S. albus*. The gums showed marked improvement, but the soreness around two or three teeth still persisted; the skin generally, from being itchy and showing a tendency to pimples, cleared up and to her great delight remained sightly and comfortable.

*Case 4.*—Mrs. A., aged about 50, came to me with all the teeth loose, complaining of great fatigue, trouble with the eyes, constipation and a steady loss of weight. In six months the vaccine treatment had markedly tightened the teeth and her chronic fatigue and constipation were gone; the wrinkles vanished from her skin owing to an increase of weight from 115 to 127. Her eyes, she says, were so improved that she could readily read what before was impossible or accomplished with difficulty.

*Case 5.*—A married woman, aged 40, who had been under my local treatment for about two years took a vaccine last fall composed of a streptococcus. She had a complexion covered with red patches, gout in the right eye, hemoglobin 67 and great depression with constant fatigue. In six months the hemoglobin went up to 93, the gout disappeared from the eye, her complexion cleared and the gums made more progress than had previously been made in two years under local treatment. Her greatest satisfaction lay in the fact that she slept well, which prior to the vaccine had not been the case.

In three cases as the gums and abscesses healed marked redness of the nose disappeared. This was associated with great clearing of the complexion and a gain in weight. In other cases there was a consistent healing of the gums that seemed much more rapid than that usually obtained from local treatment alone, but with many of them the time is too short to be sure of a permanence in cure.

In one case that refused to yield either to local or vaccine treatment a tumor of the breast was discovered. Such a depressing focus was obviously a sufficient reason for the poor results obtained, and as the patient refuses to have an operation the prognosis is not good.

Vaccine treatment judiciously used seems to be so valuable in the cases of pyorrhea that it should, in my opinion, always be given a fair trial and always in sympathetic cooperation with the family physician.—*Journal A. M. A., December 20, 1913.*

